# Improving the Efficiency of Organic Solar Cells upon Addition of Polyvinylpyridine

**DOI:** 10.3390/ma7128189

**Published:** 2014-12-22

**Authors:** Rita Rodrigues, Rui Meira, Quirina Ferreira, Ana Charas, Jorge Morgado

**Affiliations:** 1 Instituto de Telecomunicações, Avenida Rovisco Pais, P-1049-001 Lisboa, Portugal; E-Mails: rf.rodrigues@yahoo.com (R.R.); ruimeira@netcabo.pt (R.M.); quirinatf@gmail.com (Q.F.); ana.charas@ist.utl.pt (A.C.); 2 Department of Bioengineering, Instituto Superior Técnico, UL, Avenida Rovisco Pais, P-1049-001 Lisboa, Portugal

**Keywords:** polymer blends and alloys, organic photovoltaics, polyvinylpyridine

## Abstract

We report on the efficiency improvement of organic solar cells (OPVs) based on the low energy gap polyfluorene derivative, APFO-3, and the soluble C_60_ fullerene PCBM, upon addition of a residual amount of poly (4-vinylpyridine) (PVP). We find that the addition of 1% by weight of PVP with respect to the APFO-3 content leads to an increase of efficiency from 2.4% to 2.9%. Modifications in the phase separation details of the active layer were investigated as a possible origin of the efficiency increase. At high concentrations of PVP, the blend morphology is radically altered as observed by Atomic Force Microscopy. Although the use of low molecular weight additives is a routine method to improve OPVs efficiency, this report shows that inert polymers, in terms of optical and charge transport properties, may also improve the performance of polymer-based solar cells.

## 1. Introduction

In recent years the field of organic photovoltaics (OPV) has been a prolific research ground due to the promise of combining an inexpensive technology, mechanical flexibility and the prospect of integration, by using conformable materials. However, the remaining bottlenecks, in particular the poorer performance of organic photovoltaic cells when compared to the more expensive but still high performing inorganic semiconductor-based ones, are preventing this technology from gaining traction within the photovoltaic energy area [1,2].

Along with the development of new materials, significant efforts are focused on device performance improvement upon optimization of the active layer morphology, either by solvent selection, temperature and solvent annealing processes or lower molecular weight additives. Among such additives, thiols [3], and, in particular, 1,8-diiodooctane (DIO) [4] and triphenylamine (TPA) [5], have been used and shown to lead to significant performance improvements [6]. Reports indicate this effect is due to morphology modifications of the active layer, leading to active films with rougher surfaces. In such cases, the low molecular weight additives act as co-solvents, which affect domain formation during solvent evaporation. It is not clear how much of the additive remains in the active layer after the fabrication of the device is finalized, though differences in morphology are clearly observed [7]. A recent report [8] shows that the addition of an insulating polymer (polystyrene, PS) improves the performance of solar cells based on small molecules. The authors have shown that polystyrene domains are dispersed throughout the active layer, and attributed to PS a similar effect to that of DIO, by promoting the crystallization of the donor molecule. However, no comparable investigations have been made in polymer-based solar cells. 

**Figure 1:**
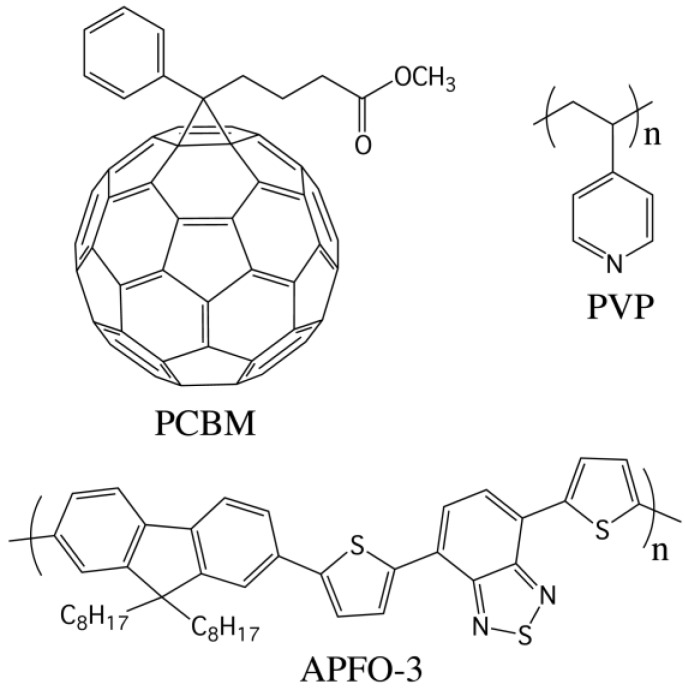
Structures of PCBM, APFO-3 and the dopant poly (4-vinylpyridine) (PVP).

Here we report on the effect of poly (4-vinylpyridine) (PVP) (Figure 1) addition to blends of poly[(9,9-dioctylfluorenyl-2,7-diyl)-*alt*-5,5-(4’,7’-di-2-thienyl-2’,1’,3’-benzothiadiazole)] (APFO-3), also known as F8TBT, with [6,6]-phenyl C61-butyric acid methyl ester (PCBM) [9,10]. We find that the addition of 1% by weight of PVP, with respect to APFO-3, leads to an increase of power conversion efficiency, PCE, from 2.4 to 2.9%. Further increase of PVP content leads to films with rather rough surface and a high number of short-circuited devices. APFO-3 was selected as a low energy gap polymer (Figure 1), being its low energy gap due to the alternating combination of electron-donor and electron-acceptor monomers, a strategy shown to be effective at extending light absorption into the infrared [11,12].

## 2. Results and Discussion

**Figure 2:**
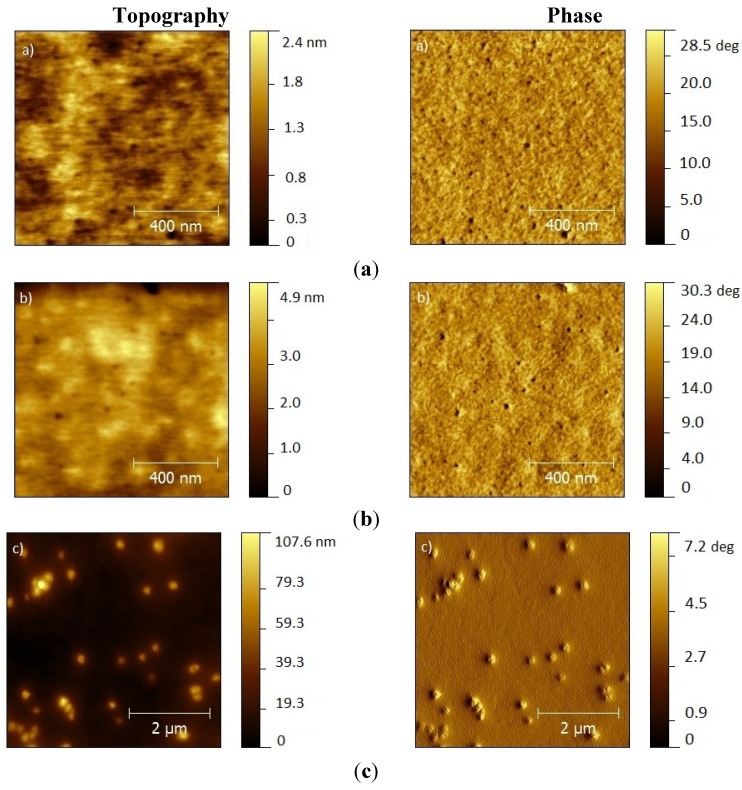

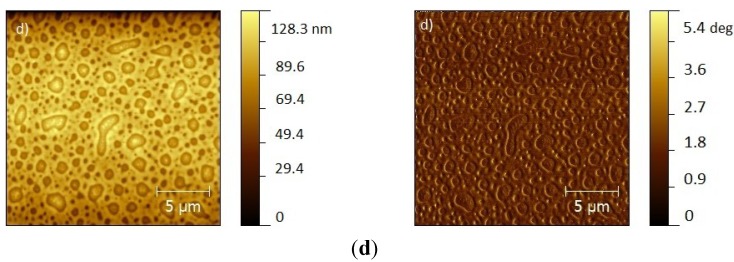
Topography (left) and phase (right) Atomic Force Microscopy (AFM) images of (**a**) regular APFO:PCBM blends; (**b**) APFO:PCBM:1% PVP blend (with 1% PVP, w/w with respect to APFO-3); (**c**) same composition as (**b**) with twice the spin-coating deposition speed; (**d**) APFO:PCBM:15% PVP blend (with 15% PVP, w/w with respect to APFO-3).

Figure 2a shows the topography and phase images of a film of APFO-3:PCBM blend and Figure 2b shows the corresponding images obtained for the APFO-3:PCBM:PVP blends films with 1% PVP (w/w with respect to APFO-3). It can be observed that, under these conditions, both films show similar surface topography and phase, without a visible effect of the addition of PVP. This is not surprising in view of the low amount of PVP. At this scale, for both cases with low amounts of PVP, the absence of color contrast in the phase images (Figure 2a,c) suggests a good combination of donor and acceptor. This is in keeping with reports in the literature of a much lower degree of phase separation of PCBM with copolymers which have the benzothiadiazole group within the polymer backbone [13] and APFO-3 lower repulsive interaction toward PCBM when compared with other copolymers [14].

The average quadratic surface roughness, RMS, obtained for both films shows little difference. For images recorded over 0.5 µm × 0.5 µm, we find that RMS increases from 0.297 nm to 0.393 nm upon addition of PVP. When the spinning speed used to deposit the film of APFO:PCBM:1% PVP is increased from 500 rpm to 1000 rpm, we find large aggregates on the surface of the film reaching heights of over 100 nm (Figure 2c) leading to short-circuited photovoltaic devices. Also, higher amounts of PVP in the active layer lead to non-functioning devices; AFM images (shown in Figure 2d) show strong evidence for phase separation for these films with higher PVP content. It has been previously reported in the literature that PVP containing blends form PVP rich areas in the film due to selective or poorer solubility when compared to the other blend components during spin-coating. Usually, these areas are elevated when compared to the rest of the film, and both polymer concentration and spinning speed contribute to this lateral separation [15,16].

**Figure 3:**
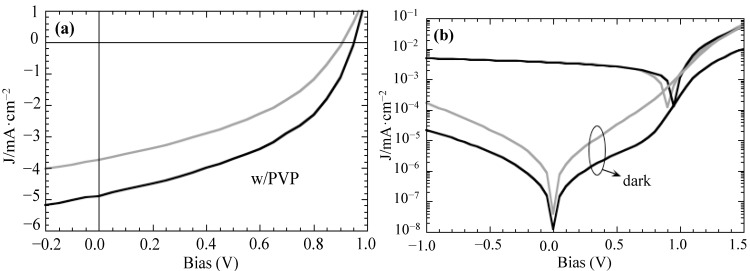
(**a**) Solar I-V characteristics for photovoltaic devices based in APFO-3:PCBM (dark grey) and APFO-3:PCBM:1%PVP (black); (**b**) solar and dark I-V characteristics on a logarithmic scale.

Figure 3a shows the current density as a function of the bias voltage of the two types of OPVs (without and with PVP) under illumination. It can be observed that the addition of PVP to APFO-3:PCBM blends increases both the open circuit voltage (V_oc_), from 0.91 to 0.95 V, and the short circuit current density (J_sc_), from 3.8 mA·cm^−2^ to 4.9 mA·cm^−2^. It is worth mentioning that the I-V characteristics of the APFO-3:PCBM heterojunction device are in observance with reported values [6,7]. Series (R_s_) and parallel (R_p_) resistances were determined from the slopes of the current-voltage curves. The addition of PVP slightly reduces both R_s_, from 69 to 51 Ω·cm^2^, and R_p_, from 833 to 667 Ω·cm^2^, leading also to a slight increase of the fill factor from 0.40 to 0.44. These results show that charge extraction is made easier upon addition of PVP, while increasing charge recombination.

The comparison of the dark and under illumination current-voltage curves in a log scale (Figure 3b) provides an additional visualization of the V_oc_ variation and shows that a better defined injection region is defined upon addition of PVP. 

It has been reported that higher efficiencies in APFO-3:PCBM blends can be obtained when spontaneously formed four-fold multilayer structures with an APFO-3 enriched free surface are present, as opposed to vertically homogeneous films [17,18]. 

The results show that not only is the beneficial effect of PVP limited to rather low content but that, even for such cases, the details of film formation are critical. At variance with the results obtained with low molecular weight additives, where some may be released during device fabrication, no doubt PVP is retained in the active layer. Based on the AFM results obtained for the best performing devices, no clear effect of the PVP presence is detected. However, this is limited to the surface analysis and additional studies of the bulk need to be carried out in order to understand the effect of PVP on the sample morphology.

As mentioned above, the injection region on the semi-log I-V plot indicates improvements between the semiconductor and the top contact when PVP is added (Figure 3b). PCBM-only photovoltaic devices were prepared to independently evaluate this effect without the possible morphology influence on the blend. Considering that the work functions of bottom and top contacts (ITO:PEDOT:PSS and LiF:Al) are the same no substantial differences are to be expected in these devices. PCBM-only photovoltaic devices present poor rectification and a high failure statistic as expected [19]. However, when PVP is present, the dark response of the devices shows good rectification with higher V_oc_ values, with an average value of 0.32 V.

Previous reports have identified the poor interface adhesion between the organic active layer and the contacts [20], when evaluating all the component interfaces in a photovoltaic device, as a limiting performance factor. The vinylpyridine monomer has been identified as an adhesion promoting agent for metal-polymer interfaces due to the strong interaction of the pyridine functional group [21,22]. In the work carried out by Huang *et al.* [8], mentioned above, it is also found that the addition of polystyrene to the blend of PCBM and the low molecular weight donor improves the adhesion. It is therefore possible that the addition of PVP may improve the adhesion between the APFO-3:PCBM layer and the top LiF/Al contact, in spite the very low PVP content.

## 3. Experimental Section

Photovoltaic devices were prepared using ITO-coated glass substrates, previously cleaned and treated with oxygen plasma, coated with a 25 nm thick layer of PEDOT:PSS (purchased from Heraeus Clevios, Leverkusen, Germany) by spin coating and heated at 125 °C for 10 min over a hotplate. The active layer comprised of a APFO-3:PCBM blend (total solids concentration of 12 mg·mL^−1^) was spin-coated from a chloroform solution (HPLC grade, Aldrich) at 1000 rpm (average thickness of 110 nm) or at 500 rpm (for the first 18 seg) when PVP (used as received from Aldrich, average *M_w_* *ca.* 160,000) was present (average thickness of 84 nm) for a total time of 80seg. APFO-3 was prepared from the monomers 2,7-bis(4,4,5,5-tetramethyl-1,3,2-dioxaborolan-2-yl)-9,9-dioctylfluorene [13] and 4,7-bis(5-bromo-2-thienyl)-2,1,3-benzothiadiazole [14] by a Suzuki coupling polymerization reaction, at 90 °C, for 72 h, using Pd(PPh_3_)_4_ (4% mol) as catalyst in EtOH_4_ aq./toluene. The polymer was precipitated in methanol and extracted with chloroform in a Soxlet apparatus. Number average (*M*_n_) and weight average molecular weight (*M*_w_), determined by Gel Permeation Chromatography (GPC) against polystyrene standards, were 8.0 kDa and 15.4 kDa, respectively. PC_60_BM was purchased from Solenne (Groningen, The Netherlands) and used as received. The photovoltaic devices were completed with a 1.5 nm thick LiF layer and an Al capping layer of *ca.* 70 nm, both deposited by thermal evaporation at a base pressure of 2 × 10^−6^ mbar. Photovoltaic characterization was made under inert atmosphere conditions. The solar response was acquired using an Oriel Instruments Solar Simulator 92250A-1000 (AM 1.5G 0.7 sun output power determined with a calibrated silicon solar cell). Atomic Force Microscopy (AFM) studies were carried out with a Molecular Imaging Agilent (model 5100) and a Nano Observer from Concept Scientific Instruments (Les Ulis, France) using non-contact mode with cantilivers having resonant frequencies between 200 kHz and 400 kHz and silicon tips of under 10nm radii. Gwydion (version 2.26) software was used for data processing.

## 4. Conclusions 

In conclusion, we have demonstrated that incorporation of PVP into the active layers of OPV devices leads to increased power conversion efficiencies (PCE), which presents a new opportunity in the OPV field. Photovoltaic characterization points to a more efficient injection process. AFM surface studies failed to provide a link between morphology and the PCE increase, but we believe that PVP presence induces a better morphology of the active layer, similar to the role of PS in small-molecule based OPV. Additionally, there may be an improved adhesion between the organic layer and the top electrode, promoted by PVP. 
